# ChatGPT and Gemini in warfarin counseling

**DOI:** 10.3325/cmj.2025.66.399

**Published:** 2025-12

**Authors:** Muhammet Hüseyin Erkan, Ömer Faruk Rahman, Abdullah Güner, Fevzi Ayyıldız, Emin Barbarus

**Affiliations:** 1 Department of Cardiovascular Surgery, Balıkesir University, Balıkesir, Turkey; 2 Department of Cardiovascular Surgery, Faculty of Medicine, Izmir Bakircay University, Izmir, Turkey; 3 Department of Cardiovascular Surgery, Konya City Hospital, Konya, Turkey; 4 Department of Cardiovascular Surgery, Afyon State Hospital, Turkish Ministry of Health, Afyonkarahisar, Turkey

## Abstract

**Aim:**

To compare the accuracy, scientific adequacy, and clarity of responses provided by ChatGPT-4o and Gemini to frequently asked patients’ questions about warfarin use.

**Methods:**

Forty patients’ questions were posed to ChatGPT and Gemini using the zero-shot method. Four cardiovascular surgeons evaluated the responses for adequacy, scientific accuracy, and clarity on a 5-point Likert scale. The first and second set of data evaluations were separated by seven days to minimize any memory effect. The experts were blinded to the type of LLM that created the response.

**Results:**

ChatGPT responses were significantly shorter (187.3 ± 47.6 vs 291.4 ± 98.1 words; *P* < 0.001) but scientifically more adequate (4.38 ± 0.30 vs 4.17 ± 0.35; *P* = 0.004). Gemini scored higher in terms of clarity (4.69 ± 0.24 vs 4.48 ± 0.33; *P* < 0.001). The two models did not significantly differ in terms of accuracy (*P* = 0.606).

**Conclusion:**

Both LLMs provide reliable and understandable information for warfarin counseling. While ChatGPT provides dense and scientifically detailed responses, Gemini excels in clarity and user-friendly communication. However, expert supervision and appropriate clinical guidance are critical for safe and comprehensive guidance.

Warfarin is a widely used oral anticoagulant that prevents thromboembolic events in atrial fibrillation, deep vein thrombosis, pulmonary embolism, and mechanical heart valve replacement. Although the ease of use and fixed-dose advantages of oral anticoagulants have increased, warfarin remains an unavoidable and often lifelong treatment option for certain patient groups. Due to its narrow therapeutic range, drug and food interactions, and the need for regular international normalized ratio (INR) monitoring, patients find warfarin complex to use and frequently search for information on its use (1).

Artificial intelligence (AI)-based large language models (LLMs) offer a new paradigm for accessing information in the health care field (2). As technology advances, trust in online platforms as sources of medical information is increasing (3). However, there are concerns regarding the accuracy, scientific validity, and transparency of the information provided by these systems (4). In particular, the extent to which AI systems can reliably address clinically critical issues remains controversial (5). However, despite these concerns, there is a paucity of evidence systematically evaluating the accuracy, scientific adequacy, and clarity of information generated by AI-based large language models in response to patients’ questions involving high-risk medications such as warfarin. This study aims to evaluate the accuracy, scientific adequacy, and clarity of responses provided by ChatGPT and Gemini to 40 frequently asked patients’ questions on warfarin use.

## MATERIAL AND METHODS

### Question pool creation

Frequently asked questions were identified through a comprehensive review of online sources, including Google Trends, YouTube search suggestions, patient support forums, and official websites of health institutions, associations, and organizations. Additionally, the research team compiled patients’ questions frequently encountered in clinical practice. Forty questions were selected and grouped into three categories: (i) usage, dosage, and monitoring; (ii) side effects, complications, and emergencies; and (iii) nutrition, drug ınteractions, and lifestyle.

### Responses and response length analysis

Free memberships were created for ChatGPT-4o (OpenAI, Microsoft Corporation, San Francisco, CA, USA; access date: July 25, 2025) and Gemini-2.5 Flash (Google, Mountain View, CA, USA; access date: July 25, 2025) using a newly created email address that had not previously been associated with any AI models. To ensure that the AI models were not influenced by prior data, they were downloaded with zero prior knowledge. For each question, a separate “new chat” session was opened in each LLM (“new chat” in ChatGPT, “start over” in Gemini). The question text was entered into the prompt field without any additional commands (zero-shot method). Each model generated responses without seeing all question-answer pairs, and contextual transfer between questions was completely eliminated. The responses for each question were recorded without any modifications, and the word counts for each question were manually recorded.

### Expert panel

The responses were evaluated by four faculty members of four different cardiovascular surgery clinics who had at least five years of expertise. The responses obtained from the two LLMs were transferred to separate Word files named “Booklet A” and “Booklet B.” No references to LLM were included. The matching information was stored only by the data analyst. At the first evaluation session, all panelists were given Booklet A by the data analyst. After a seven-day wash-out period, all panelists received Booklet B. The wash-out period was used to minimize any memory or carryover effects from the first session. The researcher who designed the study did not participate in the evaluation process in order to maintain blinding. The panelists evaluated the accuracy, scientific adequacy, and clarity of each booklet on a 5-point Likert scale (1 = very inadequate, 2 = inadequate, 3 = average, 4 = good, 5 = excellent). For the purpose of this study, accuracy was defined as the extent to which the information provided was factually correct and consistent with current evidence-based clinical guidelines. Scientific adequacy referred to the appropriateness, completeness, and clinical relevance of the information in addressing the question. Clarity was defined as the degree to which the information was presented in a clear, understandable, and patient-appropriate manner, avoiding ambiguity or unnecessary technical complexity.

The final score was calculated as the arithmetic mean of the scores given by the four experts. Operational definitions for each rating level were provided in writing to the panelists prior to scoring, and all evaluations were performed independently based on these definitions.

### Statistical analysis

The distribution of data was evaluated with the Shapiro-Wilk normality test. A dependent-samples *t* test was used to assess the differences in matched measurements. Continuous variables are expressed as mean ± standard deviation (SD). The level of statistical significance was set at *P* < 0.05. Additionally, effect sizes for the comparisons were calculated using Cohen’s d, defined as the difference between group means divided by the pooled SD, to assess the practical significance of the observed differences. The analysis was performed with SPSS, version 27 (IBM Corp., Armonk, NY, USA).

## RESULTS

Overall, both LLMs provided generally coherent and contextually relevant responses to patients’ questions; however, several qualitative limitations were identified. Potentially harmful or clearly incorrect recommendations were rare. The most common reasons for lower accuracy or scientific adequacy scores included oversimplification of complex clinical scenarios, omission of critical safety warnings (such as the need for individualized INR monitoring or physician consultation), and occasional lack of alignment with current guidelines. In some instances, responses contained ambiguous phrasing that could lead to misinterpretation by patients, particularly regarding dose adjustments and management of drug-food or drug-drug interactions. Examples included providing generalized advice without emphasizing contraindications or failing to highlight situations requiring urgent medical attention.

The questions used in the study are shown in Table 1. ChatGPT (187.3 ± 47.58 words) gave significantly shorter responses than Gemini (291.4 ± 98.11 words; *P* < 0.001). When examining overall evaluator scores, ChatGPT (4.38 ± 0.30 vs 4.17 ± 0.35) scored higher on the scientific adequacy domain (*P* = 0.004, Cohen’s d = 0.64), while Gemini scored higher on the clarity domain (4.69 ± 0.24 vs 4.48 ± 0.33; *P* < 0.001, Cohen’s d = −0.73). The models did not differ in terms of the scores in the accuracy domain (*P* > 0.17, Cohen’s d = −0.25). The average score across all domains was 4.46 ± 0.26 for ChatGPT and 4.48 ± 0.27 for Gemini (*P* = 0.606, Cohen’s d = −0.08). Subgroup analysis of LLM performance by evaluator is shown in Supplemental Table 1.(Supplementary Table 1)

**Table 1:** ChatGPT’s and Gemini’s responses to 40 warfarin-related patients’ questions

		Word count		
Question number	Question	Chat GPT	Gemini	Difference
1	Who are the candidates for warfarin therapy?	225	365	140
2	What should be the target international normalized ratio (INR) value for a patient using warfarin?	139	222	83
3	At what time of day should warfarin be taken—morning or evening?	358	179	−179
4	Should warfarin be taken on an empty stomach or with food?	167	137	−30
5	Can the dosage of warfarin be changed?	234	395	161
6	What should I do if I forget to take my warfarin dose?	162	280	118
7	What happens if I take two doses of warfarin on the same day?	166	331	165
8	My INR value is above 3; is this a cause for concern?	223	265	42
9	My INR value is 1.5; what does this indicate?	159	198	39
10	How frequently should INR monitoring be performed?	166	276	110
11	Do I need to be fasting for an INR test?	87	174	87
12	Should warfarin be used lifelong?	174	276	102
13	Does warfarin take effect immediately?	113	136	23
14	Can warfarin be used together with other anticoagulants?	234	371	137
15	Can I adjust my own warfarin dose?	153	520	367
16	Does warfarin cause frequent nosebleeds?	162	274	112
17	Does warfarin cause headaches?	211	156	−55
18	Does warfarin use cause itching?	123	202	79
19	If I experience gum bleeding, should I stop taking warfarin?	187	168	−19
20	My menstrual bleeding has increased with warfarin use; is this normal?	164	223	59
21	I developed bruises on my skin while taking warfarin; what could be the cause?	197	413	216
22	Can a minor fall while on warfarin cause internal bleeding?	213	366	153
23	How should warfarin be discontinued if emergency surgery is required?	208	463	255
24	What should I do if I need a tooth extraction while taking warfarin?	194	339	145
25	Does long-term use of warfarin damage the organs?	233	423	190
26	Which foods should be avoided while taking warfarin?	247	434	187
27	Can I consume green leafy vegetables such as spinach, arugula, or parsley while on warfarin?	162	259	97
28	Does warfarin interact with fruits such as grapefruit or pomegranate?	149	241	92
29	Can I drink herbal tea while taking warfarin?	173	365	192
30	Can I take fish oil or omega-3 supplements while using warfarin?	166	234	68
31	Is it safe to take vitamin or mineral supplements while on warfarin?	231	402	171
32	Can I consume alcohol while taking warfarin?	191	353	162
33	Is caffeine (tea/coffee) consumption a problem for warfarin users?	202	241	39
34	Does smoking affect INR levels?	208	322	114
35	Should I inform other physicians about my warfarin use if they prescribe medication?	110	150	40
36	Can women using warfarin become pregnant?	173	297	124
37	Does warfarin affect sexual function?	157	285	128
38	Can I engage in sports while taking warfarin?	224	408	184
39	Can I drive while taking warfarin?	202	203	1
40	Should warfarin dosage be adjusted before air travel?	245	310	65
	Mean ± standard deviation	187.3 ± 47.58	291.4 ± 98.11	
	*P* value			<0.001
	Test statistic			−7.114

## DISCUSSION

In our study, ChatGPT was superior to Gemini in terms of scientific accuracy, presenting its responses with more concise and dense content. This finding suggests that the model may have a knowledge-intensive yet clear communication strategy. In contrast, Gemini’s responses were more explanatory and user-friendly. In terms of accuracy, both models performed similarly, indicating that both systems can provide reliable information in a basic advisory context.

In similar evaluations conducted in different clinical areas, LLMs’ responses to patients’ questions are mostly rated as “good” or higher and can provide useful content from a practical standpoint when used carefully (6-8). However, some studies emphasize that the current levels of accuracy and source transparency are insufficient for these models to be adopted as a primary source of patient information and cannot replace personalized physician-patient communication (9,10).

Although the use of LLMs in health care is rapidly becoming widespread, some concerns remain. For example, scoping reviews indicate that LLMs have significant potential in patient note generation, rare disease diagnosis, and clinical scenario presentation, but emphasize that human oversight is indispensable with a “human-in-the-loop” approach (11). Additionally, retrieval-augmented generation (RAG) mechanisms have been shown to improve the accuracy, completeness, and safety performance of LLMs. Indeed, the RAG-supported Almanac model achieved significantly higher accuracy rates in clinical scenarios (12). Although our study did not directly integrate RAG, ChatGPT and Gemini were able to generate highly accurate responses.

LLM-based clinical decision support systems are successfully applied in the context of drug safety. RAG-supported LLMs perform better in detecting drug-related errors than LLM-based approaches alone, and the best results are achieved in the co-pilot mode (expert + LLM collaboration) (13). Similarly, a study examining the accuracy of patient instructions for three different medications showed that ChatGPT potentially offers high accuracy but carries the risk of misguidance due to incomplete content (14).

Additionally, ChatGPT-4 aligns well with current clinical guidelines on diet, medication, and anticoagulation management in pre-colonoscopy counseling, but social biases and the risk of “hallucinations” persist (15). Notably, ChatGPT-4's rate of complete, accurate information in tests on atrial fibrillation management increased from 45% in 2023 to 73% in 2024 (16). This increase demonstrates that models' clinical competence can be improved through continuous updating and training.

Differences between the models in this study, although statistically significant, may be quite small when evaluated on a 5-point scale and may have limited practical significance. The fact that both models scored in the “good” to “excellent” range suggests that the statistical differences may not translate into noticeable performance distinctions in actual clinical practice. However, the content differences observed in individual questions reveal the strengths and weaknesses of the models. For example, a striking content difference emerged between the two models in question 15: “Can I adjust my warfarin dosage myself?” ChatGPT responded to the question with a clear “absolutely not,” drawing a strict line in terms of patient safety, but did not mention programs such as self-monitoring (PST) and self-management (PSM), which require special training and are recommended for specific patient groups in international guidelines. High-level evidence shows that PSM significantly reduces the risk of complications, lowers mortality, and has been proven safe (17-19). On the other hand, Gemini stated that the dose should not be directly adjusted, but explained in detail that PST/PSM programs can be applied to trained patients under certain specific conditions, that INR measurement and algorithm-based dose adjustment can be performed at home, but only in selected patient groups, under physician supervision, and after structured training. While this approach is richer in content, it also carries a clinical risk as it can be misinterpreted by some users if not properly framed.

This example demonstrates that models should be evaluated not only by average scores but also by the quality of their responses to critical clinical questions. Evaluating the content quality, safety, and scope of responses using more qualitative and quantitative tests, rather than solely scoring them on a Likert scale, can yield more concrete information about the clinical suitability of models. For example, a study using GPT-4 evaluated corporate heart failure patient education materials using multiple readability tests (the Flesch Reading Ease score, Flesch-Kincaid Grade Level, Gunning Fog Index, Coleman-Liau Index, Simple Measure of Gobbledygook Index, and Automated Readability Index). After revision by the model, both readability and comprehensiveness of the material were significantly improved (20). Such multidimensional evaluation methods more comprehensively reveal content quality and clinical safety. However, in our study, the models' responses were only scored using a Likert scale, and the lack of more comprehensive qualitative tests in terms of content quality and clinical accuracy is a limitation.

The LLM responses evaluated in this study provide general and standard recommendations; however, they cannot take into account patient-specific parameters and risk profiles. For example, responses provided with a zero-shot approach cannot account for individualized risk factors such as age, comorbidities, concomitant medication use, and bleeding history. The accuracy of LLMs is limited when structured guidance or explicit instructions are not provided, and they may potentially compromise patient safety (21). Therefore, LLM-based responses should only be used for general informational purposes; it is unsafe to substitute them for expert opinion in personalized clinical practice. While this demonstrates the potential benefits of LLMs, it also suggests that they may be limited in personalized anticoagulation management.

This study has some limitations. First, the research was conducted using only two large language models and based on responses obtained at a single point in time. As these models are constantly updated, their performance and accuracy levels may change over time. Second, the responses were evaluated by four cardiovascular surgery experts, and there is a possibility of subjective evaluation bias. Including more diverse groups of evaluators (health care professionals from different specialties and patients) could yield different rating scores. Third, although the question pool was selected based on a systematic content analysis of various online sources and clinical experience, the questions may not represent all patient concerns; this may limit the generalizability of the study. Furthermore, the study did not aim to achieve complete agreement among evaluators, and inter-rater reliability was not reported due to the subjective expert-based evaluation of LLM responses; this limitation was considered appropriate for the methodological purpose of the study. In this study, the models' responses were only scored using a Likert scale, and the lack of more comprehensive qualitative tests in terms of content quality and clinical accuracy is a limitation. Finally, the models' responses were evaluated only in terms of accuracy, scientific adequacy, and clarity; potential safety risks were not systematically classified. Therefore, the possibility that some responses could be clinically misinterpreted or pose a risk was not considered. Future studies are recommended to add a safety classification such as “safe/unsafe” for each response to enable a more comprehensive evaluation of the models in terms of patient safety.

In conclusion, warfarin management is an individualized and complex process; LLM responses only provide general information and cannot replace individualized clinical decisions. Incomplete or misinterpreted responses could pose serious risks, particularly in situations such as specialized self-management programs. Therefore, LLM-based systems should only be used under the supervision of licensed health care professionals and should never be relied upon as the sole basis for treatment decisions. The findings reveal that AI-powered information resources can serve as a valuable complementary tool in patient education and health communication.
