# Supplementary material for: ChatGPT and Gemini in warfarin counseling
Source: Croat Med J. 2025 Dec;66(6):399–405. doi: 10.3325/cmj.2025.66.399 (PMC12856749; doi:10.3325/cmj.2025.66.399)
Supplement: Supplementary Table 1 [file CroatMedJ_66_s005.pdf]

Supplemental Table 1. Comparison of GPT-4o and Gemini Scores Across Reviewers and Domains

	Chat-GPT	Gemini	test stat	p value	Cohen's d
Reviewer1(Accuracy)	4.47 ± 0.75	4.40 ± 0.74	0,476	0,637	0.094
Reviewer1(Science)	3.98 ± 0.58	4.07 ± 0.53	-0,781	0,44	-0.162
Reviewer1(Clarity)	4.03 ± 0.86	4.42 ± 0.68	-2,726	**0,01**	-0.503
Reviewer2(Accuracy)	4.72 ± 0.45	4.83 ± 0.39	-1,275	0,21	-0.261
Reviewer2(Science)	4.07 ± 0.57	4.38 ± 0.59	-2,926	**0,006**	-0.534
Reviewer2(Clarity)	4.43 ± 0.59	4.58 ± 0.50	-1,29	0,205	-0.274
Reviewer3(Accuracy)	4.07 ± 0.42	4.17 ± 0.64	-0,85	0,401	-0.185
Reviewer3(Science)	4.33 ± 0.53	3.93 ± 0.57	3,252	**0,002**	0.727
Reviewer3(Clarity)	4.55 ± 0.60	4.90 ± 0.30	-3,557	**0,001**	-0.738
Reviewer4(Accuracy)	4.75 ± 0.49	4.95 ± 0.22	-2,449	**0,019**	-0.527
Reviewer4(Science)	4.45 ± 0.50	4.30 ± 0.46	1,356	0,183	0.312
Reviewer4(Clarity)	4.93 ± 0.27	4.88 ± 0.34	0,813	0,421	0.163
Overall(Accuracy)	4.51 ± 0.31	4.59 ± 0.34	-1,378	0,176	-0.246
Overall(Science)	4.38 ± 0.30	4.17 ± 0.35	3,102	**0,004**	0.644
Overall(Clarity)	4.48 ± 0.33	4.69 ± 0.24	-3,823	**<0,001**	-0.728
Overall(MeanScore)	4.46 ± 0.26	4.48 ± 0.27	-0,519	0,606	-0.075
